# Synthesis and crystal structure of methyl 3-(3-hy­droxy-3-phenyl­prop-2-eno­yl)benzoate

**DOI:** 10.1107/S2056989018007259

**Published:** 2018-05-18

**Authors:** Irina S. Zharinova, Alfiya A. Bilyalova, Stanislav I. Bezzubov

**Affiliations:** a Department of Chemistry, Lomonosov Moscow State University, Lenin’s Hills 1/3, Moscow 119991, Russian Federation; b Kurnakov Institute of General and Inorganic Chemistry, Russian Academy of Sciences, Leninskiy pr. 31, Moscow 119991, Russian Federation

**Keywords:** crystal structure, *β*-diketone, hydrogen bond, tautomerism

## Abstract

The title compound, C_17_H_14_O_4_, was synthesized under mild conditions and characterized by various analytical techniques. Combined NMR and X-ray diffraction data show that the substance exists exclusively in the enol tautomeric form. An intra­molecular ⋯O=C—C=C—OH⋯ hydrogen bond is present in the mol­ecular structure. The analysis of the difference density map disclosed two adjacent positions of a disordered hydrogen atom taking part in this hydrogen bond, indicating the presence of two enol tautomers in the crystal. The enol mol­ecules are assembled through numerous C—H⋯π and π–π as well as weak C(ar­yl)—H⋯O inter­actions, thus forming a dense crystal packing. The obtained substance was also studied by UV–Vis spectroscopy and cyclic voltammetry.

## Abstract 2

A non-symmetric aromatic *β*-diketone enol bearing a carb­oxy­methyl group has been synthesized and characterized by X-ray crystallography, ^1^H and ^13^C NMR spectroscopy, elemental analysis, UV–Vis spectroscopy and cyclic voltammetry.

## Chemical context   

The high complexing ability *via* O-donor atoms and excellent optical properties of aromatic *β*-diketones make them practically irreplaceable in the creation of efficient emitters [as lanthanide or iridium(III) complexes] for application in OLEDs (organic light-emitting diodes; Eliseeva & Bünzli, 2010 ▸; Bünzli, 2015 ▸). In addition, *β*-diketone-based Ir^III^ complexes have attracted particular attention as promising photosensitizers in dye-sensitized solar cells (Baranoff *et al.*, 2010 ▸). Surprisingly, aromatic *β*-diketones functionalized by anchoring COOH groups have not been considered as a possible alternative to traditional anchoring 4,4′-dicarb­oxy-2,2′-bi­pyridine groups.

Herein we report on the crystal structure as well as optical and electrochemical properties of a non-symmetric aromatic *β*-diketone with formula C_17_H_14_O_4_, bearing a carb­oxy­methyl group.
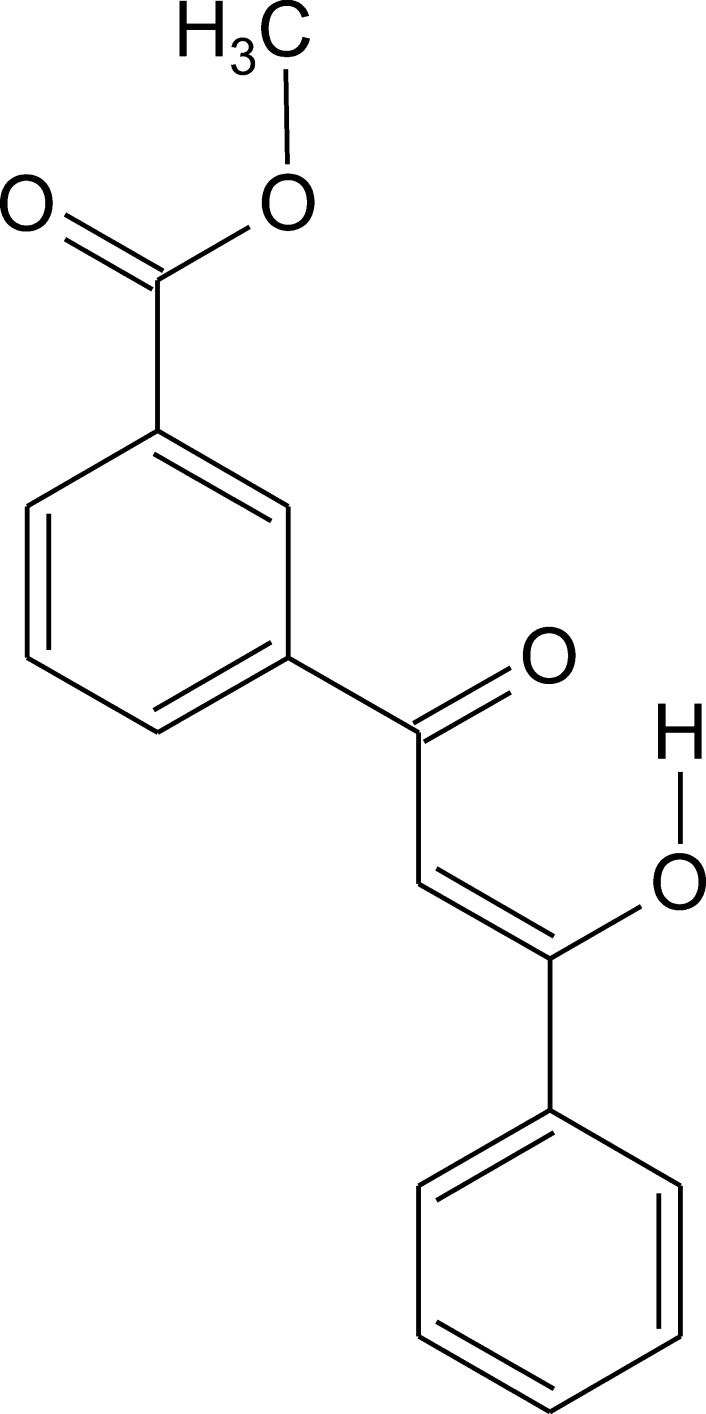


## Structural commentary   

**Figure 1:**
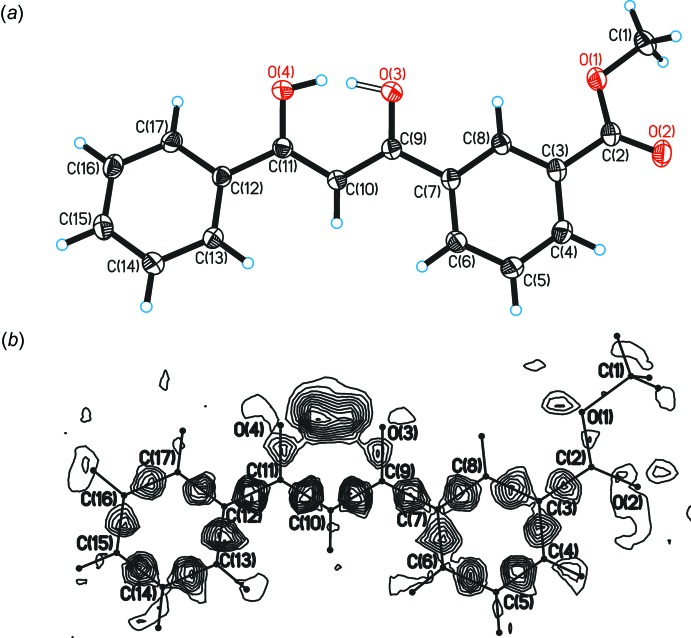
(*a*) Mol­ecular structure of 3-(3-hy­droxy-3-phenyl­prop-2-eno­yl)benzoate. Displacement ellipsoids are shown at the 50% probability level; (*b*) difference-density map in the plane of the hydrogen-bonded ring. This map was computed after least-squares refinement without the hydrogen atoms H21 and H22 involved in the hydrogen bond. Contours are drawn at 0.04 e Å^−3^ inter­vals.

A ^1^H NMR study of the prepared *β*-diketone showed that it appears exclusively as an enol tautomer in solution (CDCl_3_). Single-crystal X-ray diffraction analysis also confirmed unambiguously that the compound exists in the enol form in the solid state (Fig. 1 ▸ *a*). In the mol­ecular structure, an intra­molecular resonance-assisted hydrogen bond (for related structures, see: Gilli *et al.*, 2004 ▸) connects the two oxygen atoms of the keto–enol moiety with the O3⋯O4 distance as short as 2.4358 (10) Å (Table 1 ▸). The hydrogen atom involved in this inter­action is disordered over two sites (H21 and H22) with almost equal occupancies. The virtual H⋯H distance of 0.625 (1) Å is a result of the simultaneous presence of two enol forms, O3—H⋯O4 and O3⋯H—O4, respectively, in an approximate 1:1 ratio in the crystal. The title mol­ecule is almost planar with a variation of the dihedral angles between phenyl rings and the keto–enol plane between 5.65 (4) and 11.05 (4)°.

## Supra­molecular features   

**Figure 2:**
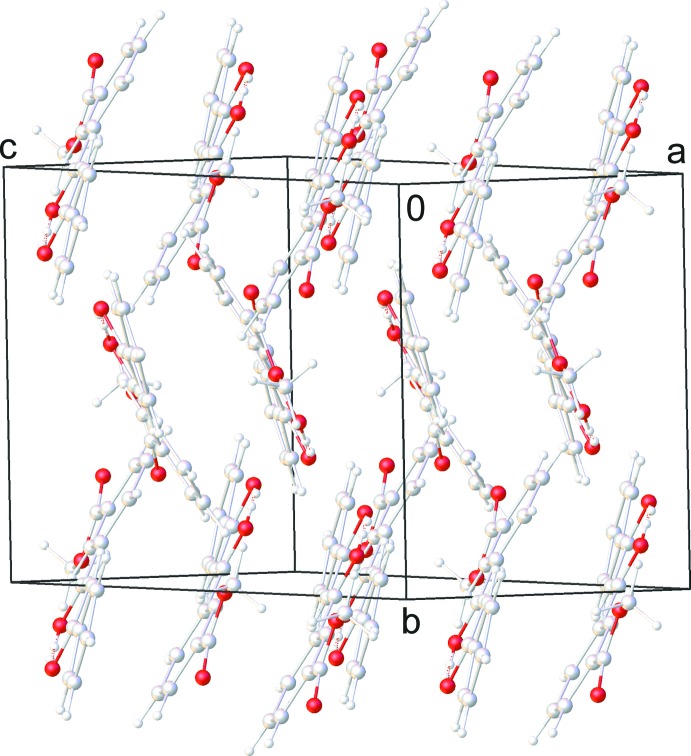
Crystal packing of 3-(3-hy­droxy-3-phenyl­prop-2-eno­yl)benzoate.

The enol mol­ecules are assembled in a ‘head-to-tail’ manner by several C—H⋯π [range 2.740 (15)–2.758 (15) Å] inter­actions (Table 1 ▸]) involving the phenyl H atoms and the centroids of the phenyl rings of adjacent mol­ecules as well as by π–π contacts [range 3.422 (14)–3.531 (15) Å]. The resultant stacks are grafted together by weak C—H⋯O inter­actions (Desiraju & Steiner, 2001 ▸) between the aryl rings and the oxygen atoms of the keto–enol fragment with a C⋯O distance of 3.0837 (12) Å, forming a network structure (Table 1 ▸; Figs. 2 ▸ and 3 ▸).

## Database survey   

Although there have been numerous reports on crystal structures of various symmetric and non-symmetric *β*-diketones in the Cambridge Structural Database (Version 5.38, February 2018; Groom *et al.*, 2016 ▸), only a few examples of aromatic *β*-diketones functionalized by COOH groups (or COO*R*) are well documented (Langer *et al.*, 2006 ▸; Ishikawa & Ugai, 2013 ▸; Hui *et al.*, 2010 ▸). In their mol­ecular structures, the intra­molecular resonance-assisted hydrogen bonds exhibit quite short O⋯O distances (2.39–2.55 Å; Bertolasi *et al.*, 1991 ▸). The hydrogen atom located between these O atoms is either ordered or disordered by symmetry as in di­benzoyl­methane and other symmetrical *β*-diketones (see, for example: Thomas *et al.*, 2009 ▸; Andrews *et al.*, 2014 ▸) or with unequal occupancies in the vast majority of non-symmetric enols (see, for instance: Aromí *et al.*, 2002 ▸, Soldatov *et al.*, 2003 ▸). In some cases, crystals contain two different enol mol­ecules (O—H⋯O and O⋯H—O) with ordered H atoms (Mohamed *et al.*, 2015 ▸; Zheng *et al.*, 2009 ▸; Bertolasi *et al.*, 1991 ▸).

## Synthesis and crystallization   

There are some synthetic difficulties encountered in preparation of carboxyl­ated *β*-diketones according to the common Claisen condensation. Fortunately, the desired compounds can be obtained under mild conditions *via* an MgBr_2_·Et_2_O-assisted acyl­ation of ketones by benzotriazole amides of the corresponding diesters (Lim *et al.*, 2007 ▸). The title compound was prepared as follows:

To a suspension of MgBr_2_·Et_2_O (0.73 g, 2.8 mmol) in dry CH_2_Cl_2_ (16 ml), aceto­phenone (0.35 ml, 3.0 mmol) was added and the mixture was sonicated for a minute. *N*,*N*-Diiso­propyl­ethyl­amine (0.52 ml, 3.0 mmol) was added to the mixture and it was sonicated for a minute. The resulted suspension was added quickly to a solution of the methyl ester of isophtalic acid benzotriazole amide (1.15 g, 4.0 mmol) in dry CH_2_Cl_2_ (16 ml) and the mixture was stirred at 293 K for 34 h. The reaction mixture was treated by a 2 *M* HCl solution (40 ml) and stirred vigorously for 1 h. The organic layer was separated and the aqueous layer extracted with CH_2_Cl_2_ (3 × 20 ml). The combined organic extracts were washed with water (1 × 20 ml) and brine (1 × 20 ml) and filtrated through paper followed by evaporation of the solvent. The resulting oil was crystallized from CH_3_OH solution at 255 K to give a light-yellow powder, which was purified by column chromatography (SiO_2_, CHCl_3_/hexane 1/3 *v*/*v*) and dried *in vacuo*. Yield 457 mg (54%). Single crystals suitable for X-ray analysis were grown by slow evaporation of the solvent from a solution of the substance in chloro­form.

Analysis: calculated for C_17_H_14_O_4_: C, 72.33; H, 5.00. Found: C, 72.28; H, 5.04.

^1^H NMR (CDCl_3_, ppm, 400 MHz): *δ* 3.99 (*s*, 3H, CH_3_), 6.92 (*s*, 1H, C–H), 7.51 (*t*, *J* = 7.5 Hz, 2H, Ar–H), 7.57–7.62 (*m*, 2H, Ar–H), 8.02 (d, J = 7.4 Hz, 2H, Ar–H), 8.22 (*t*, *J* = 7.8 Hz, 2H, Ar–H), 8.63 (*s*, 1H, Ar–H). See supplementary Fig. S1.

^13^C NMR (CDCl_3_, ppm, 100 MHz): *δ* 51.97, 92.81, 126.85, 127.78, 128.29, 128.50, 130.29, 130.96, 132.27, 132.74, 134.82, 135.45, 165.88, 183.99, 185.71. See supplementary Fig. S2.

UV–Vis (CH_2_Cl_2_): λ_max_ = 344 nm (∊_max_ = 32000 cm^−1^ M^−1^). See supplementary Fig. S3.

Redox potentials (Ar-saturated CH_3_CN with 0.01 *M* (*n*-Bu_4_N)ClO_4_ at scan rate of 25 mV s^−1^, ferrocene as external standard): *E*_ox1_ = 1.15, *E*_ox2_ = 1.53 V *vs* standard hydrogen electrode. See supplementary Fig. S4.

## Refinement   

Crystal data, data collection and structure refinement details are summarized in Table 2 ▸. All hydrogen atoms were located from a difference-density map and refined freely. The disordered hydrogen atoms H21 and H22 were clearly discernible from a difference-density map (Fig. 1 ▸ *b*). Their occupancies refined to a ratio of 0.44 (7):0.56 (7) and with *U*_iso_(H) = 1.5*U*_eq_(O).

## Supplementary Material

Crystal structure: contains datablock(s) I. DOI: 10.1107/S2056989018007259/wm5445sup1.cif

Structure factors: contains datablock(s) I. DOI: 10.1107/S2056989018007259/wm5445Isup2.hkl

Supporting information file. DOI: 10.1107/S2056989018007259/wm5445Isup3.mol

^1^H-NMR spectrum of 3-(3-hydroxy-3-phenylprop-2-enoyl)benzoate.. DOI: 10.1107/S2056989018007259/wm5445sup4.tif

^13^C-NMR spectrum of 3-(3-hydroxy-3-phenylprop-2-enoyl)benzoate.. DOI: 10.1107/S2056989018007259/wm5445sup5.tif

UV-Vis spectrum of 3-(3-hydroxy-3-phenylprop-2-enoyl)benzoate in CH2Cl2 at 298 K.. DOI: 10.1107/S2056989018007259/wm5445sup6.tif

Polarographic curves of 3-(3-hydroxy-3-phenylprop-2-enoyl)benzoate (CH3CN, 298 K).. DOI: 10.1107/S2056989018007259/wm5445sup7.tif

Supporting information file. DOI: 10.1107/S2056989018007259/wm5445Isup8.cml

CCDC reference: 1838743

Additional supporting information:  crystallographic information; 3D view; checkCIF report

## Figures and Tables

**Figure 3:**
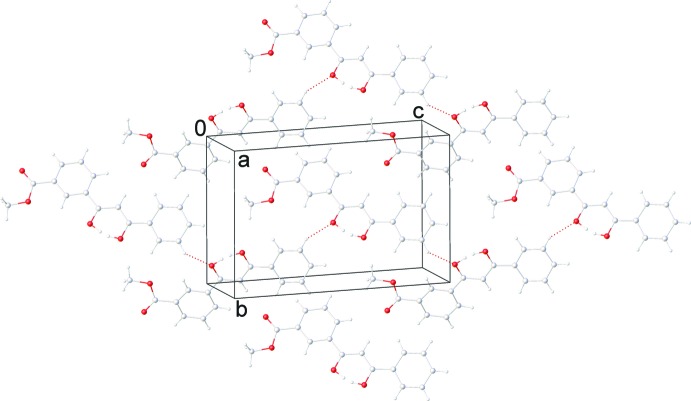
Inter­molecular C—H⋯O hydrogen bonding in the crystal structure of 3-(3-hy­droxy-3-phenyl­prop-2-eno­yl)benzoate.

**Table 1:** Hydrogen-bond geometry (Å, °) *Cg*1 and *Cg*2 are the centroids of the C3–C8 and C12–C17 rings, respectively.

*D*—H⋯*A*	*D*—H	H⋯*A*	*D*⋯*A*	*D*—H⋯*A*
O3—H21⋯O4	0.92 (7)	1.54 (7)	2.4358 (10)	162 (4)
O4—H22⋯O3	0.94 (6)	1.55 (6)	2.4358 (10)	156 (3)
C16—H16⋯O3^i^	0.956 (14)	2.417 (14)	3.0837 (12)	126.6 (11)
C5—H5⋯*Cg*2^ii^	0.990 (14)	2.740 (15)	3.525 (13)	135.0 (8)
C14—H14⋯*Cg*1^iii^	0.990 (14)	2.758 (15)	3.968 (12)	127.2 (8)

Symmetry codes: (i) 
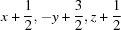
; (ii) 
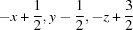
; (iii) 
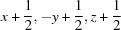
.

**Table 2:** Experimental details

Crystal data	
Chemical formula	C_17_H_14_O_4_
*M*_r_	282.28
Crystal system, space group	Monoclinic, *P*2_1_/*n*
Temperature (K)	150
*a*, *b*, *c* (Å)	7.8085 (10), 10.5171 (14), 17.124 (2)
β (°)	102.711 (2)
*V* (Å^3^)	1371.8 (3)
*Z*	4
Radiation type	Mo *K*α
μ (mm^−1^)	0.10
Crystal size (mm)	0.40 × 0.40 × 0.40

Data collection	
Diffractometer	Bruker SMART APEXII
Absorption correction	Multi-scan (*SADABS*; Bruker, 2008 ▸)
No. of measured, independent and observed [*I* > 2σ(*I*)] reflections	16222, 4006, 3488
*R*_int_	0.019
(sin θ/λ)_max_ (Å^−1^)	0.703

Refinement	
*R*[*F* ^2^ > 2σ(*F* ^2^)], *wR*(*F* ^2^), *S*	0.039, 0.114, 1.03
No. of reflections	4006
No. of parameters	249
H-atom treatment	Only H-atom coordinates refined
Δρ_max_, Δρ_min_ (e Å^−3^)	0.37, −0.22

Computer programs: *APEX2* and *SAINT* (Bruker, 2008 ▸), *SHELXL2014/7* (Sheldrick, 2015 ▸), *SHELXTL* (Sheldrick, 2008 ▸), *OLEX2* (Dolomanov *et al.*, 2009 ▸) and *publCIF* (Westrip, 2010 ▸).

## supplementary crystallographic information

### Crystal data

**Table:**

C_17_H_14_O_4_	*F*(000) = 592
*M**_r_* = 282.28	*D*_x_ = 1.367 Mg m^−^^3^
Monoclinic, *P*2_1_/*n*	Mo *K*α radiation, λ = 0.71073 Å
*a* = 7.8085 (10) Å	Cell parameters from 7383 reflections
*b* = 10.5171 (14) Å	θ = 2.3–30.6°
*c* = 17.124 (2) Å	µ = 0.10 mm^−^^1^
β = 102.711 (2)°	*T* = 150 K
*V* = 1371.8 (3) Å^3^	Block, colorless
*Z* = 4	0.40 × 0.40 × 0.40 mm

### Data collection

**Table:**

Bruker SMART APEXII diffractometer	3488 reflections with *I* > 2σ(*I*)
Radiation source: fine-focus sealed tube	*R*_int_ = 0.019
ω scans	θ_max_ = 30.0°, θ_min_ = 2.3°
Absorption correction: multi-scan (*SADABS*; Bruker, 2008)	*h* = −10→10
	*k* = −14→14
16222 measured reflections	*l* = −24→23
4006 independent reflections	

### Refinement

**Table:**

Refinement on *F*^2^	0 restraints
Least-squares matrix: full	Hydrogen site location: difference Fourier map
*R*[*F*^2^ > 2σ(*F*^2^)] = 0.039	Only H-atom coordinates refined
*wR*(*F*^2^) = 0.114	*w* = 1/[σ^2^(*F*_o_^2^) + (0.0663*P*)^2^ + 0.2977*P*] where *P* = (*F*_o_^2^ + 2*F*_c_^2^)/3
*S* = 1.03	(Δ/σ)_max_ = 0.001
4006 reflections	Δρ_max_ = 0.37 e Å^−^^3^
249 parameters	Δρ_min_ = −0.22 e Å^−^^3^

### Special details

**Table:**

Geometry. All esds (except the esd in the dihedral angle between two l.s. planes) are estimated using the full covariance matrix. The cell esds are taken into account individually in the estimation of esds in distances, angles and torsion angles; correlations between esds in cell parameters are only used when they are defined by crystal symmetry. An approximate (isotropic) treatment of cell esds is used for estimating esds involving l.s. planes.

### Fractional atomic coordinates and isotropic or equivalent isotropic displacement parameters (Å^2^)

**Table:**

	*x*	*y*	*z*	*U*_iso_*/*U*_eq_	Occ. (<1)
O1	−0.02289 (10)	0.46985 (7)	0.30296 (4)	0.02697 (17)	
O2	−0.12474 (11)	0.26937 (7)	0.28859 (4)	0.03277 (19)	
O3	0.38132 (11)	0.60581 (7)	0.53783 (4)	0.02964 (17)	
H21	0.453 (8)	0.656 (5)	0.576 (4)	0.044*	0.44 (7)
O4	0.57483 (11)	0.70032 (7)	0.65449 (4)	0.02985 (18)	
H22	0.514 (6)	0.679 (3)	0.602 (3)	0.045*	0.56 (7)
C1	−0.10630 (15)	0.49402 (11)	0.22003 (6)	0.0300 (2)	
C2	−0.04506 (12)	0.35314 (9)	0.32993 (5)	0.02290 (19)	
C3	0.03831 (11)	0.33830 (9)	0.41682 (5)	0.02148 (18)	
C4	−0.00756 (13)	0.23323 (10)	0.45741 (6)	0.0255 (2)	
C5	0.06741 (13)	0.21642 (10)	0.53842 (6)	0.0272 (2)	
C6	0.18868 (12)	0.30374 (9)	0.57898 (6)	0.02360 (19)	
C7	0.23487 (11)	0.40975 (8)	0.53865 (5)	0.01984 (17)	
C8	0.15825 (12)	0.42696 (9)	0.45750 (5)	0.02100 (18)	
C9	0.35890 (12)	0.50819 (8)	0.57990 (5)	0.02039 (18)	
C10	0.44676 (12)	0.49939 (8)	0.66080 (5)	0.02051 (18)	
C11	0.55464 (12)	0.59996 (8)	0.69575 (5)	0.02053 (18)	
C12	0.65178 (12)	0.60014 (8)	0.78042 (5)	0.01982 (17)	
C13	0.66742 (13)	0.49140 (9)	0.82826 (6)	0.02281 (19)	
C14	0.76486 (13)	0.49531 (10)	0.90678 (6)	0.0260 (2)	
C15	0.84439 (13)	0.60801 (10)	0.93823 (6)	0.0264 (2)	
C16	0.82857 (13)	0.71666 (10)	0.89115 (6)	0.0266 (2)	
C17	0.73336 (13)	0.71309 (9)	0.81247 (6)	0.02385 (19)	
H1	−0.232 (2)	0.4767 (14)	0.2115 (9)	0.039 (4)*	
H2	−0.085 (2)	0.5860 (16)	0.2101 (9)	0.049 (4)*	
H3	−0.0581 (19)	0.4386 (14)	0.1844 (8)	0.036 (3)*	
H4	−0.0921 (19)	0.1711 (14)	0.4287 (8)	0.036 (3)*	
H5	0.0327 (18)	0.1435 (14)	0.5681 (8)	0.036 (3)*	
H6	0.2389 (17)	0.2880 (12)	0.6355 (8)	0.028 (3)*	
H8	0.1889 (17)	0.4998 (13)	0.4304 (8)	0.030 (3)*	
H10	0.4322 (18)	0.4265 (13)	0.6917 (8)	0.028 (3)*	
H13	0.6130 (18)	0.4132 (13)	0.8092 (8)	0.031 (3)*	
H14	0.7750 (18)	0.4169 (13)	0.9395 (8)	0.034 (3)*	
H15	0.9105 (19)	0.6135 (14)	0.9943 (9)	0.038 (4)*	
H16	0.8836 (18)	0.7951 (13)	0.9107 (8)	0.034 (3)*	
H17	0.7219 (18)	0.7909 (13)	0.7788 (8)	0.035 (3)*	

### Atomic displacement parameters (Å^2^)

**Table:**

	*U*^11^	*U*^22^	*U*^33^	*U*^12^	*U*^13^	*U*^23^
O1	0.0310 (4)	0.0272 (4)	0.0201 (3)	−0.0005 (3)	−0.0001 (3)	−0.0007 (3)
O2	0.0381 (4)	0.0291 (4)	0.0263 (4)	−0.0026 (3)	−0.0033 (3)	−0.0062 (3)
O3	0.0382 (4)	0.0254 (4)	0.0218 (3)	−0.0056 (3)	−0.0012 (3)	0.0055 (3)
O4	0.0421 (4)	0.0227 (3)	0.0224 (3)	−0.0083 (3)	0.0019 (3)	0.0034 (3)
C1	0.0315 (5)	0.0358 (5)	0.0202 (4)	0.0036 (4)	0.0003 (4)	0.0018 (4)
C2	0.0208 (4)	0.0251 (4)	0.0221 (4)	0.0024 (3)	0.0032 (3)	−0.0035 (3)
C3	0.0194 (4)	0.0241 (4)	0.0205 (4)	0.0027 (3)	0.0035 (3)	−0.0031 (3)
C4	0.0227 (4)	0.0266 (5)	0.0265 (5)	−0.0031 (3)	0.0039 (3)	−0.0033 (4)
C5	0.0270 (5)	0.0273 (5)	0.0269 (5)	−0.0045 (4)	0.0055 (4)	0.0016 (4)
C6	0.0236 (4)	0.0259 (4)	0.0207 (4)	0.0000 (3)	0.0037 (3)	0.0010 (3)
C7	0.0186 (4)	0.0211 (4)	0.0193 (4)	0.0018 (3)	0.0030 (3)	−0.0015 (3)
C8	0.0206 (4)	0.0222 (4)	0.0197 (4)	0.0018 (3)	0.0033 (3)	−0.0010 (3)
C9	0.0204 (4)	0.0204 (4)	0.0200 (4)	0.0017 (3)	0.0035 (3)	−0.0004 (3)
C10	0.0234 (4)	0.0192 (4)	0.0182 (4)	−0.0011 (3)	0.0029 (3)	0.0000 (3)
C11	0.0227 (4)	0.0196 (4)	0.0194 (4)	0.0003 (3)	0.0049 (3)	−0.0007 (3)
C12	0.0209 (4)	0.0198 (4)	0.0188 (4)	−0.0013 (3)	0.0044 (3)	−0.0025 (3)
C13	0.0240 (4)	0.0201 (4)	0.0229 (4)	−0.0024 (3)	0.0022 (3)	−0.0004 (3)
C14	0.0256 (5)	0.0274 (5)	0.0234 (4)	−0.0011 (3)	0.0018 (4)	0.0027 (3)
C15	0.0248 (4)	0.0320 (5)	0.0208 (4)	−0.0019 (4)	0.0015 (3)	−0.0037 (4)
C16	0.0283 (5)	0.0259 (5)	0.0250 (4)	−0.0056 (4)	0.0044 (4)	−0.0074 (4)
C17	0.0283 (4)	0.0202 (4)	0.0233 (4)	−0.0031 (3)	0.0062 (3)	−0.0028 (3)

### Geometric parameters (Å, º)

**Table:**

O1—C2	1.3361 (12)	C7—C8	1.3985 (12)
O1—C1	1.4486 (12)	C7—C9	1.4858 (13)
O2—C2	1.2127 (12)	C8—H8	0.953 (13)
O3—C9	1.2881 (11)	C9—C10	1.4072 (12)
O3—H21	0.92 (7)	C10—C11	1.4017 (12)
O4—C11	1.2989 (11)	C10—H10	0.952 (13)
O4—H22	0.94 (6)	C11—C12	1.4811 (12)
C1—H1	0.980 (15)	C12—C13	1.3963 (13)
C1—H2	1.003 (17)	C12—C17	1.4015 (12)
C1—H3	0.977 (14)	C13—C14	1.3922 (13)
C2—C3	1.4952 (12)	C13—H13	0.950 (14)
C3—C4	1.3936 (14)	C14—C15	1.3904 (14)
C3—C8	1.3945 (13)	C14—H14	0.990 (14)
C4—C5	1.3932 (14)	C15—C16	1.3882 (14)
C4—H4	0.981 (15)	C15—H15	0.986 (15)
C5—C6	1.3903 (14)	C16—C17	1.3887 (13)
C5—H5	0.990 (14)	C16—H16	0.956 (14)
C6—C7	1.3999 (13)	C17—H17	0.993 (14)
C6—H6	0.975 (13)		
			
C2—O1—C1	115.82 (8)	C7—C8—H8	119.3 (8)
C9—O3—H21	101 (3)	O3—C9—C10	120.41 (8)
C11—O4—H22	103 (2)	O3—C9—C7	116.37 (8)
O1—C1—H1	109.5 (9)	C10—C9—C7	123.21 (8)
O1—C1—H2	106.3 (9)	C11—C10—C9	119.19 (8)
H1—C1—H2	110.7 (13)	C11—C10—H10	120.3 (8)
O1—C1—H3	110.8 (8)	C9—C10—H10	120.5 (8)
H1—C1—H3	108.0 (12)	O4—C11—C10	120.96 (8)
H2—C1—H3	111.5 (12)	O4—C11—C12	115.73 (8)
O2—C2—O1	123.67 (9)	C10—C11—C12	123.31 (8)
O2—C2—C3	124.07 (9)	C13—C12—C17	119.40 (8)
O1—C2—C3	112.25 (8)	C13—C12—C11	122.22 (8)
C4—C3—C8	119.94 (8)	C17—C12—C11	118.37 (8)
C4—C3—C2	118.40 (8)	C14—C13—C12	120.07 (8)
C8—C3—C2	121.65 (8)	C14—C13—H13	117.7 (8)
C5—C4—C3	119.97 (9)	C12—C13—H13	122.2 (8)
C5—C4—H4	120.3 (8)	C15—C14—C13	120.12 (9)
C3—C4—H4	119.8 (8)	C15—C14—H14	121.2 (8)
C6—C5—C4	120.29 (9)	C13—C14—H14	118.7 (8)
C6—C5—H5	119.2 (8)	C16—C15—C14	120.11 (9)
C4—C5—H5	120.4 (8)	C16—C15—H15	118.5 (8)
C5—C6—C7	120.05 (9)	C14—C15—H15	121.4 (8)
C5—C6—H6	117.7 (8)	C15—C16—C17	120.09 (9)
C7—C6—H6	122.2 (8)	C15—C16—H16	122.1 (8)
C8—C7—C6	119.53 (8)	C17—C16—H16	117.8 (8)
C8—C7—C9	118.28 (8)	C16—C17—C12	120.20 (9)
C6—C7—C9	122.15 (8)	C16—C17—H17	120.1 (8)
C3—C8—C7	120.22 (9)	C12—C17—H17	119.7 (8)
C3—C8—H8	120.5 (8)		

### Hydrogen-bond geometry (Å, º)

Cg1 and Cg2 are the centroids of the C3–C8 and C12–C17 rings, respectively.

**Table:**

*D*—H···*A*	*D*—H	H···*A*	*D*···*A*	*D*—H···*A*
O3—H21···O4	0.92 (7)	1.54 (7)	2.4358 (10)	162 (4)
O4—H22···O3	0.94 (6)	1.55 (6)	2.4358 (10)	156 (3)
C16—H16···O3^i^	0.956 (14)	2.417 (14)	3.0837 (12)	126.6 (11)
C5—H5···*Cg*2^ii^	0.990 (14)	2.740 (15)	3.525 (13)	135.0 (8)
C14—H14···*Cg*1^iii^	0.990 (14)	2.758 (15)	3.968 (12)	127.2 (8)

Symmetry codes: (i) *x*+1/2, −*y*+3/2, *z*+1/2; (ii) −*x*+1/2, *y*−1/2, −*z*+3/2; (iii) *x*+1/2, −*y*+1/2, *z*+1/2.
