# Supplementary figures and images for: Synthesis and crystal structure of methyl 3-(3-hy­droxy-3-phenyl­prop-2-eno­yl)benzoate
Source: Acta Crystallogr E Crystallogr Commun. 2018 May 18;74(Pt 6):816–9. doi: 10.1107/S2056989018007259 (PMC6002825; doi:10.1107/S2056989018007259)

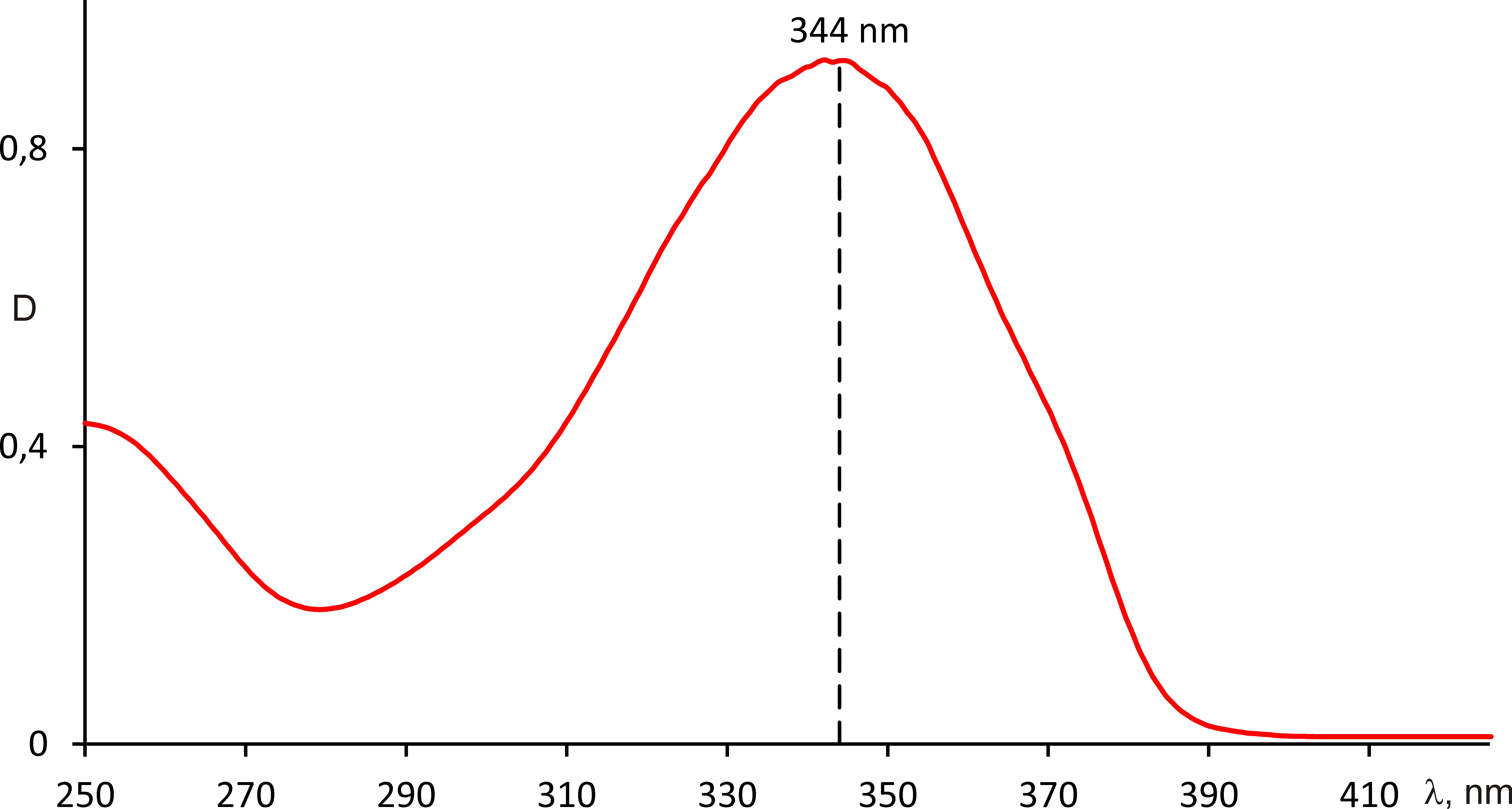

Supplement: Supplementary file 6 [file e-74-00816-sup6.tif]

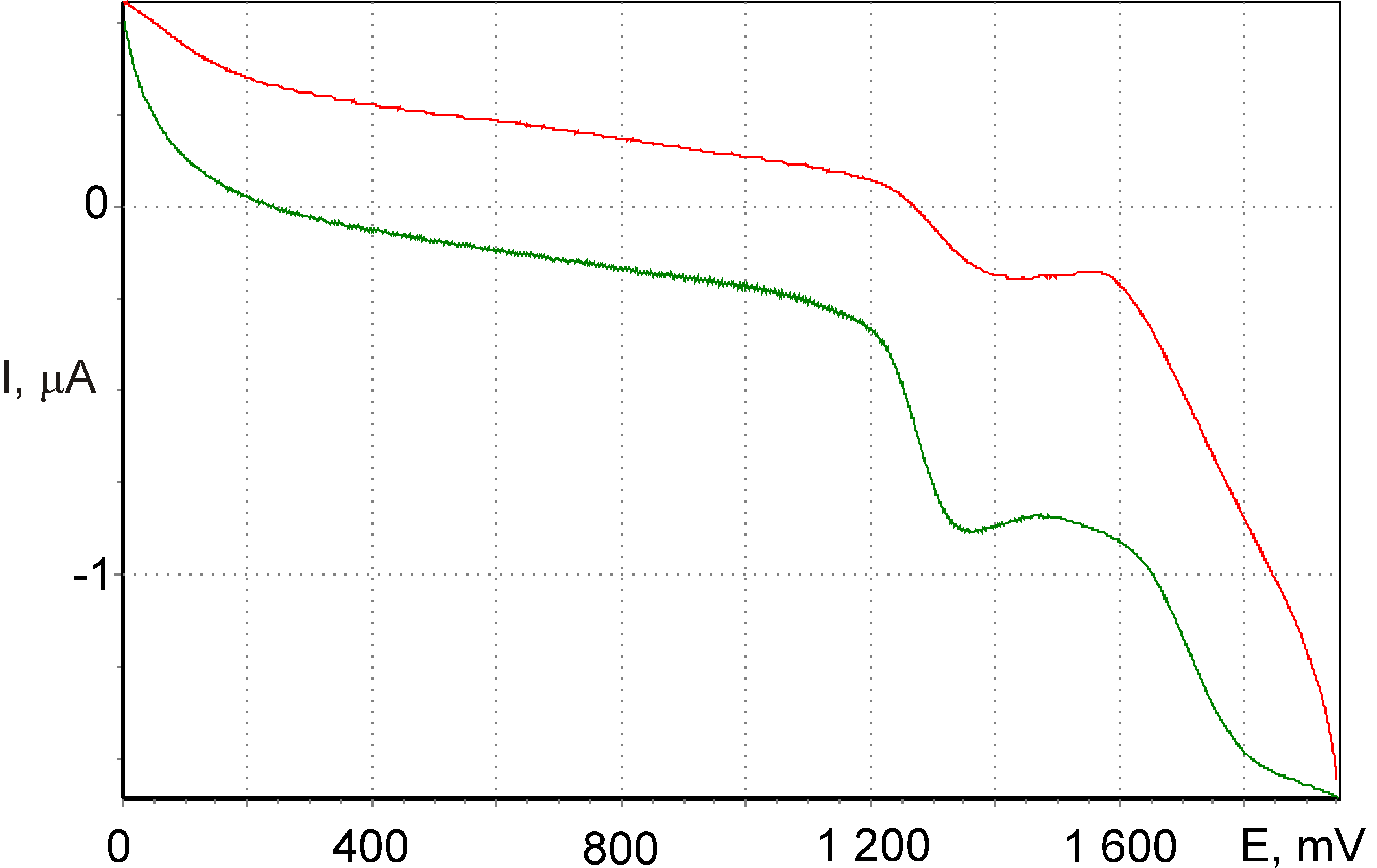

Supplement: Supplementary file 7 [file e-74-00816-sup7.tif]
